# Haplotype breeding for unlocking and utilizing plant genomics data

**DOI:** 10.3389/fgene.2022.1006288

**Published:** 2022-11-15

**Authors:** Mayank Rai, Wricha Tyagi

**Affiliations:** School of Crop Improvement, College of Post Graduate Studies, Central Agricultural University (Imphal), Imphal, Meghalaya, India

**Keywords:** alleles, genetic structure, trait, genome, haplotype

## Introduction

Next-generation sequencing technologies have made it feasible to generate large amounts of data per plant genome, and now it is even possible to generate long reads for large and complex genomes as well (Amarasinghe et al., 2020; Lan et al., 2017). Many plant genera, multiple genotypes, and many species have been sequenced (Schatz et al., 2014; Golicz et al., 2016; Zhao et al., 2020). This, in turn, has resulted in the reference genome (the genome used for the subsequent alignment of other genotypes of the same species) and pangenome (alignment of all possible representatives of different species of a particular genus) (Tettelin et al., 2021; Morgante et al., 2007) availability across multiple plant species. Some such crops are barley (Jayakodi et al., 2020), maize (Lu et al., 2015), rice (Qin et al., 2021), and soybean (Li et al., 2014; Liu et al., 2020). The next step is to utilize this information such that targeted gains for traits of importance can be achieved. This, in turn, would require a shift from the predominant approach of molecular breeding (which essentially targets a few genes/loci by making use of markers that target desired alleles) to haplotype breeding (wherein a set of alleles across loci are identified and selected simultaneously).

## Haplotype-based utilization of genomic data

Climate change and population increase are the two main factors driving the demand for progressive increase in agricultural productivity. Stress matrix data on different combinations of environmental conditions have already suggested significant negative impact on agricultural production (Mittler and Blumwald, 2010). This, in turn, puts pressure on limited genetic and land resources available for increasing agriculture production and productivity (Anderson et al., 2020). One way to address this gap is to utilize the emerging genomic data across various crops in a way that the genetic variation under selection can be best understood and then effectively selected upon for breeding-improved crops.

**FIGURE 1:**
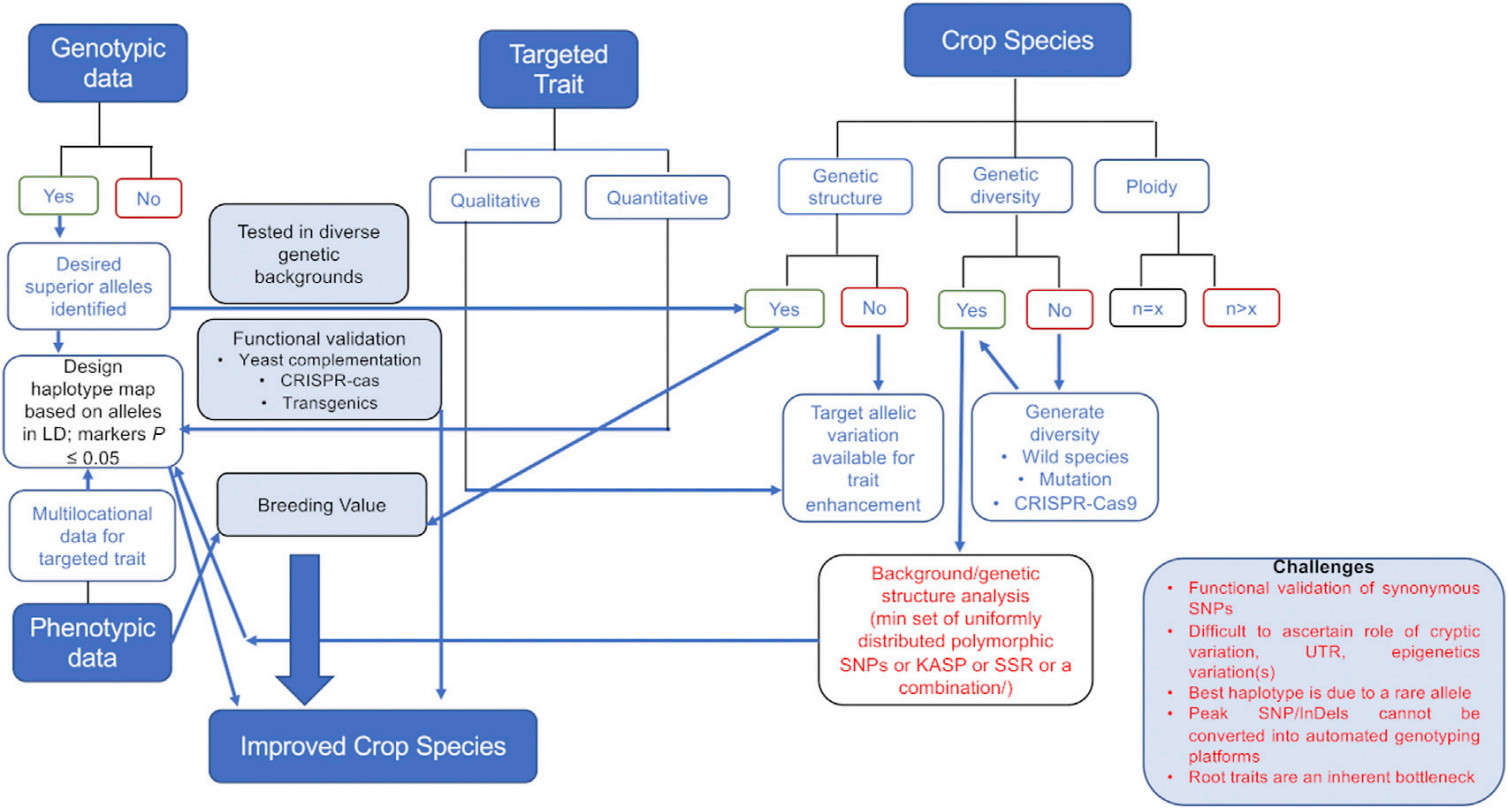
Decision-making tree involved in using genomics data for crop improvement. Genotypic data will lead to selection of significant marker(s) in the desired haplotype. The selection of a marker and haplotype will depend on the genetic structure, diversity available in the cultivated gene pool, and ploidy of the crop. Selected marker haplotypes along with the breeding value estimation of a particular genotype would help achieve genetic gains for the trait of interest. The challenges (mentioned in the box) in generating the data (root traits) and utilizing the variation (cryptic variation, rare alleles, synonymous SNPs, etc.) that still exists are also mentioned.

The attempts to enhance the genetic gain across any plant species would require the understanding of the genetic structure of that species. This means the number of sub-population groups, genetic diversity available in the cultivated gene pool, ploidy, etc., for every species need to be looked into along with the genomic information available such that utilization of the available information is incorporated while designing breeding programs targeting desired haplotypes (Figure 1).

The earliest suggestion of identifying haplotype tag SNPs (htSNPs) within haplotype-based blocks came after the human genome sequence was available with an idea of reducing the number of markers needed to capture useful information within a genomic region (Daly et al., 2001). Developing “hapmap” was suggested as a key way of understanding diseases in humans (Couzin, 2002). However, it was soon realized that the inherent genetic structure understanding of various sub-population groups was crucial (Gabriel et al., 2002), and the concept of common haplotypes emerged. The common haplotype approach has the problem of common and rare alleles being over-represented and under-represented, respectively. Pritchard and Cox (2002) suggested that this would unlikely be a problem if common diseases are caused by common variants. Therefore, traits governed by common variants/alleles can use significantly associated htSNPs once they are identified, irrespective of the population structure.

In plants, genome-wide association studies (GWAS) with diverse genotypes/populations emerged as one of the key strategies for unlocking genetic diversity (Ersoz et al., 2007). However, analyzing variants with minor or rare alleles still poses a problem due to cut-off criteria set for selection of markers. Minor allele frequencies ≤5% and markers with a missing rate higher than 10% are not considered in most studies. Also, a better understanding of differences (Tibbs Cortes et al., 2021) that arise when different GWAS methods are used is also needed. With the emergence of the concept of genomic prediction (Spindel et al., 2016; Crossa et al., 2017; Xu et al., 2020), the determination of the breeding value of an individual genotype (determined by average performance of its progeny) is now being suggested as the best way to decide selections in a breeding program. Studies in rice (a self-pollinated crop with a distinct genetic structure) have revealed that GWAS along with pedigree data and genomic selection could be effective in increasing the efficiency in breeding (Spindel et al., 2016) and that accuracy of genomic prediction was higher in less structured populations (Guo et al., 2014). If majority of the genetic variation under selection is governed by multiple small additive loci, genomic prediction and breeding value estimation would be simpler as long as an appropriate population size and the number of markers for the target species are taken into account. It is proposed that targeting ∼1 SNP every 0.2 cM (∼6–7 K SNPs) will be ideal for performing genomic selection in rice (Spindel et al., 2015). In an out-crossing crop like maize that is rich in transposons, the requirement of markers will be more as linkage disequilibrium (LD) decays much faster. As it is costly to screen large collections for specific traits of breeding interest (Holbrook and Stalker, 2003), subsets (in the form of core and mini-core collections) that represent the genetic diversity are currently being created, evaluated, and characterized for various traits across plant species (Krishnamurthy et al., 2003; Chamberlin et al., 2010; Upadhyaya et al., 2012; Schläppi et al., 2017). SoySNP50K, Illumina MaizeSNP50 Bead-Chip, and SNP data on 44,100 markers for 346 accessions of soybean, 273 accessions of maize, and 352 accessions of rice, respectively, when used to calculate pairwise SNP LD decay among these crops, revealed that the decay of LD to the *r* ^2^ = 0.25 level was much faster in maize (1 kb) than in soybean (150 kb in euchromatic and 5 kb in heterochromatic regions) or rice (123 kb) (Kaler et al., 2022). The study also revealed that prediction accuracy was the greatest for all crops when using a subset of markers that were significant at *p* ≤ 0.05. Moreover, subsets of markers selected based on the LD level did not show any change in accuracy.

## Applications of haplotype for trait enhancement in various species

Although the sequencing costs have drastically reduced, obtaining genomic data through resequencing with a high genome coverage or *de novo* assembly in crops with complex genomes for hundreds of individuals is still beyond the reach of individual research groups (Xu et al., 2020). Research groups supported by various institutes have come forward and have been working toward having open-source datasets available in the public domain. In polyploid species, the genomic data generated should be able to identify alleles distinct from the contributing parental genomes, and variants of these well-annotated previously ideal haplotypes can be discussed. In an allotetraploid species like groundnut, which lacks an available reference genome, the target enrichment sequencing approach has been applied to identify SNPs and generate haplotype-based markers for developing a genotyping platform (Peng et al., 2017; Clevenger et al., 2018). Studies in rice have revealed that the performance of an allele varies widely across different genetic backgrounds. For example, PSTOL1 (Phosphorus Starvation Tolerance 1) introgressed from the aus-type sub-group into diverse genetic backgrounds behaves differently across genetic backgrounds (Wissuwa et al., 2015), and superior haplotypes other than those originally reported are now known (Pariasca-Tanaka et al., 2014; Yumnam et al., 2017). Some of the key genes for yield and grain quality have been analyzed in detail across a 3 K rice panel, and desirable haplotypes for multiple traits have been identified with a purpose of enhancing genetic gain through haplotype selection (Abbai et al., 2019). Complex quantitative traits including yield would require an in-depth understanding of the various component traits in diverse germplasm before functionally desirable haplotypes emerge, which can be incorporated into a breeding program. It is important that molecular basis/functionality (especially in the case of cryptic genetic variations) of the desired haplotype selected be ascertained, and markers with a higher prediction accuracy be identified. In case the crop has a narrow genetic base, wild-crosses followed by 1–2 generation of backcross must be attempted, and this breeding material can then serve as a source of novel haplotypes with minimum noise (linkage drag). In species where hybridization barriers exist, *de novo* domestication by genome editing by targeting multiple genes that control desired traits simultaneously can be attempted (Pramanik et al., 2021). In the case of post-fertilization barriers, by the use of coupled haploid induction and gene editing, it is now possible to generate transgene-free and gene-edited haploids (He et al., 2022). Still, challenges like marker design for SNPs/indels which do not meet the basic quality parameter of multiplexing (lying in the hypervariable region or underlying the “predicted” pseudogenes) exist. Also, understanding the role of rare alleles, cryptic variations (UTRs/epigenetic variations), codon bias underlying synonymous SNPs having a role in the efficiency and accuracy of gene transcription and translation, etc., implies that further understanding of nucleotide variation is needed to target its usage in crop improvement programs (Figure 1). Improvement for below ground traits is an ongoing challenge. Although tremendous progress has been made in understanding root-related traits and root–soil/root–microbe interaction, a lot more is yet to be understood. The emergence of concept of practical haplotype graph (PHG), which uses a graph of haplotypes to represent the variability in a breeding program and can merge genotypes from whole-genome sequencing and marker technologies, has led to successful utilization of large genomic datasets in plants like sorghum (Jensen et al., 2020) and maize (Franco et al., 2020).

## Conclusion

The major challenge in the utilization of large-scale genomics data is to understand the variation and then target it for crop improvement programs. This, in turn, requires simultaneous identification and selection of superior allelic combinations across loci or haplotype(s) for targeting trait enhancement. The phenotypic and genotypic data available for multiple locations and diverse genotypes, respectively, have to be sieved into two parts: 1) explaining breeding value and 2) number of loci underlying a trait. Components of variations observed explaining breeding value of a trait, and haplotype needs to be clearly dissected and then targeted for marker development and deployment such that desired haplotype(s) can be fixed as early as possible in the targeted genetic background in a breeding cycle. To achieve this, genomic data currently available and being generated need to be looked from a genetics perspective of the target trait and crop species. The progress made in understanding traits (simple/complex, types of inter/intra-genic interactions, etc.) and crops (domestication history, ploidy, pollination, etc.) will have to be leveraged to make trait-specific mini-core/core collections or practical haplotype graphs with suitable marker sets available for selection of the “ideal” haplotype. The design of markers having a higher prediction value and the use of only a significantly associated subset of markers in prediction and selection will ensure that genotyping costs are not prohibitive. These can then be used by crop improvement programs targeting a particular trait.

## References

[B1] Abbai R. Singh V. K. Nachimuthu V. V. Sinha P. Selvaraj R. Vipparla A. K. (2019). Haplotype analysis of key genes governing grain yield and quality traits across 3K RG panel reveals scope for the development of tailor‐made rice with enhanced genetic gains. Plant Biotechnol. J. 17 (8), 1612–1622. 10.1111/pbi.13087 30701663 PMC6662101

[B2] Amarasinghe S. L. Su S. Dong X. Zappia L. Ritchie M. E. Gouil Q. (2020). Opportunities and challenges in long-read sequencing data analysis. Genome Biol. 21, 30. 10.1186/s13059-020-1935-5 32033565 PMC7006217

[B3] Anderson R. Bayer P. E. Edwards D. (2020). Climate change and the need for agricultural adaptation. Curr. Opin. Plant Biol. 56, 197–202. 10.1016/j.pbi.2019.12.006 32057694

[B5] Chamberlin K. D. C. Melouk H. A. Payton M. E. (2010). Evaluation of the US peanut mini core collection using a molecular marker for resistance to Sclerotinia minor Jagger. Euphytica 172, 109–115. 10.1007/s10681-009-0065-7

[B6] Clevenger J. P. Korani W. Ozias-Akins P. Jackson S. (2018). Haplotype-based genotyping in polyploids. Front. Plant Sci. 9, 564. 10.3389/fpls.2018.00564 29755500 PMC5932196

[B7] Couzin J. (2002). Genomics. New mapping project splits the community. Science 296, 1391–1393. 10.1126/science.296.5572.1391 12029111

[B8] Crossa J. Pe´rez-Rodrı´guez P. Cuevas J. Montesinos-Lo´pez O. Jarquı´n D. de los Campos G. (2017). Genomic selection in plant breeding: Methods, models, and perspectives. Trends Plant Sci. 22, 961–975. 10.1016/j.tplants.2017.08.011 28965742

[B9] Daly M. J. Rioux J. D. Schaffner S. F. Hudson T. J. Lander E. S. (2001). High-resolution haplotype structure in the human genome. Nat. Genet. 29 (2), 229–232. 10.1038/ng1001-229 11586305

[B10] Ersoz E. S. Yu J. Buckler E. S. (2007). “Applications of linkage disequilibrium and association mapping in crop plants,” in Genomics-assisted crop improvement. Editors Varshney R. K. Tuberosa R. (Dordrecht, Netherlands: Springer), 97–119.

[B11] Franco J. A. V. Gage J. L. Bradbury P. J. Johnson L. C. Miller Z. R. Buckler E. S. (2020). A maize practical haplotype graph leverages diverse NAM assemblies. bioRxiv. 10.1101/2020.08.31.268425

[B12] Gabriel S. B. Schaffner S. F. Nguyen H. Moore J. M. Roy J. Blumenstiel B. (2002). The structure of haplotype blocks in the human genome. Science 296, 2225–2229. 10.1126/science.1069424 12029063

[B13] Golicz A. A. Bayer P. E. Barker G. C. Edger P. P. Kim H. Martinez P. A. (2016). The pangenome of an agronomically important crop plant *Brassica oleracea* . Nat. Commun. 7, 13390. 10.1038/ncomms13390 27834372 PMC5114598

[B14] Guo Z. Tucker D. Basten C. Gandhi H. Ersoz E. Guo B. (2014). The impact of population structure on genomic prediction in stratified populations. Theor. Appl. Genet. 127, 749–762. 10.1007/s00122-013-2255-x 24452438

[B15] He Y. Mudgett M. Zhao Y. (2022). Advances in gene editing without residual transgenes in plants. Plant Physiol. 188 (4), 1757–1768. 10.1093/plphys/kiab574 34893903 PMC8968301

[B16] Holbrook C. C. Stalker H. T. (2003). “Peanut breeding and genetic resources,” in Plant breeding reviews. Editor Janick J. (NY: John Wiley & Sons).

[B17] Jayakodi M. Padmarasu S. Haberer G. Bonthala V. S. Gundlach H. Monat C. (2020). The barley pan-genome reveals the hidden legacy of mutation breeding. Nature 588, 284–289. 10.1038/s41586-020-2947-8 33239781 PMC7759462

[B18] Jensen S. E. Charles J. R. Muleta K. Bradbury P. J. Casstevens T. Deshpande S. P. (2020). A sorghum practical haplotype graph facilitates genome‐wide imputation and cost‐effective genomic prediction. Plant Genome 13 (1), e20009. 10.1002/tpg2.20009 33016627 PMC12807297

[B19] Kaler A. S. Purcell L. C. Beissinger T. Gillman J. D. (2022). Genomic prediction models for traits differing in heritability for soybean, rice, and maize. BMC Plant Biol. 22 (1), 87–11. 10.1186/s12870-022-03479-y 35219296 PMC8881851

[B20] Krishnamurthy L. Kashiwagi J. Upadhyaya H. D. Serraj R. (2003). Genetic diversity of drought-avoidance root traits in the mini-core germplasm collection of chickpea. Int. Chickpea Pigeonpea Newsl. (10), 21–24.

[B21] Lan T. Renner T. Ibarra-Laclette E. Farr K. M. Chang T. -H. Cervantes-Pérez S. A. (2017). Long-read sequencing uncovers the adaptive topography of a carnivorous plant genome. Proc. Natl. Acad. Sci. U. S. A. 114, E4435–E4441. 10.1073/pnas.1702072114 28507139 PMC5465930

[B22] Li Y. H. Zhou G. Ma J. Jiang W. Jin L. G. Zhang Z. (2014). De novo assembly of soybean wild relatives for pan-genome analysis of diversity and agronomic traits. Nat. Biotechnol. 32, 1045–1052. 10.1038/nbt.2979 25218520

[B23] Liu Y. Du H. Li P. Shen Y. Peng H. Liu S. (2020). Pan-genome of wild and cultivated soybeans. Cell. 182, 162–176.e13. 10.1016/j.cell.2020.05.023 32553274

[B24] Lu F. Romay M. C. Glaubitz J. C. Bradbury P. J. Elshire R. J. Wang T. (2015). High-resolution genetic mapping of maize pan-genome sequence anchors. Nat. Commun. 6, 6914. 10.1038/ncomms7914 25881062 PMC4411285

[B25] Mittler R. Blumwald E. (2010). Genetic engineering for modern agriculture: Challenges and perspectives. Annu. Rev. Plant Biol. 61 (1), 443–462. 10.1146/annurev-arplant-042809-112116 20192746

[B26] Morgante M. De Paoli E. Radovic S. (2007). Transposable elements and the plant pan-genomes. Curr. Opin. Plant Biol. 10, 149–155. 10.1016/j.pbi.2007.02.001 17300983

[B27] Pariasca-Tanaka J. Chin J. H. Drame K. N. Dalid C. Heur S. Wissuwa M. (2014). A novel allele of the P-starvation tolerance gene OsPSTOL1 from African rice (*Oryza glaberrima* Steud) and its distribution in the genus Oryza. Theor. Appl. Genet. 127, 1387–1398. 10.1007/s00122-014-2306-y 24728072 PMC4035548

[B28] Peng Z. Fan W. Wang L. Paudel D. Leventini D. Tillman B. L. (2017). Target enrichment sequencing in cultivated peanut (*Arachis hypogaea* L.) using probes designed from transcript sequences. Mol. Genet. Genomics 292 (5), 955–965. 10.1007/s00438-017-1327-z 28492983

[B29] Pramanik D. Shelake R. M. Kim M. J. Kim J. Y. (2021). CRISPR-mediated engineering across the central dogma in plant biology for basic research and crop improvement. Mol. Plant 14 (1), 127–150. 10.1016/j.molp.2020.11.002 33152519

[B30] Pritchard J. K. Cox N. J. (2002). The allelic architecture of human disease genes: Common disease–common variant or not? Hum. Mol. Genet. 11, 2417–2423. 10.1093/hmg/11.20.2417 12351577

[B31] Qin P. Lu H. Du H. Wang H. Chen W. Chen Z. (2021). Pan-genome analysis of 33 genetically diverse rice accessions reveals hidden genomic variations. Cell. 184, 3542–3558.e16. e3516. 10.1016/j.cell.2021.04.046 34051138

[B32] Schatz M. C. Maron L. G. Stein J. C. Wences A. H. Gurtowski J. Biggers E. (2014). Whole genome de novo assemblies of three divergent strains of rice, *Oryza sativa*, document novel gene space of aus and indica. Genome Biol. 15, 506. 10.1186/PREACCEPT-2784872521277375 25468217 PMC4268812

[B33] Schläppi M. R. Jackson A. K. Eizenga G. C. Wang A. Chu C. Shi Y. (2017). Assessment of five chilling tolerance traits and GWAS mapping in rice using the USDA mini-core collection. Front. Plant Sci. 8, 957. 10.3389/fpls.2017.00957 28642772 PMC5463297

[B34] Spindel J. Begum H. Akdemir D. Virk P. Collard B. Redon˜a E. (2015). Genomic selection and association mapping in rice (*Oryza sativa*): Effect of trait genetic architecture, training population composition, marker number and statistical model on accuracy of rice genomic selection in elite, tropical rice breeding lines. PLoS Genet. 11, e1004982. 10.1371/journal.pgen.1004982 25689273 PMC4334555

[B35] Spindel J. E. Begum H. Akdemir D. Collard B. Redon˜a E. Jannink J. -L. (2016). Genome-wide prediction models that incorporate de novo GWAS are a powerful new tool for tropical rice improvement. Heredity 116, 395–408. 10.1038/hdy.2015.113 26860200 PMC4806696

[B36] Tettelin H. Masignani V. Cieslewicz M. J. Donati C. Medini D. Ward N. L. (2021). Genome analysis of multiple pathogenic isolates of *Streptococcus agalactiae*: Implications for the microbial “pangenome”. Proc. Natl. Acad. Sci. U. S. A. 102, 13950–13955. 10.1073/pnas.0506758102 PMC1216834 16172379

[B37] Tibbs Cortes L. Zhang Z. Yu J. (2021). Status and prospects of genome-wide association studies in plants. Plant Genome 14, e20077. 10.1002/tpg2.20077 33442955 PMC12806871

[B38] Upadhyaya H. D. Mukri G. Nadaf H. L. Singh S. (2012). Variability and stability analysis for nutritional traits in the mini core collection of peanut. Crop Sci. 52, 168–178. 10.2135/cropsci2011.05.0248

[B39] Wissuwa M. Kondo K. Fukuda T. Mori A. Rose M. T. Pariasca-Tanaka J. (2015). Unmasking novel loci for internal phosphorus utilization efficiency in rice germplasm through genome-wide association analysis. PLoS One 10, e0124215. 10.1371/journal.pone.0124215 25923470 PMC4414551

[B40] Xu Y. Liu X. Fu J. Wang H. Wang J. Huang C. (2020). Enhancing genetic gain through genomic selection: From livestock to plants. Plant Commun. 1, 100005. 10.1016/j.xplc.2019.100005 33404534 PMC7747995

[B41] Yumnam J. S. Rai M. Tyagi W. (2017). Allele mining across two low-P tolerant genes PSTOL1 and PupK20-2 reveals novel haplotypes in rice genotypes adapted to acidic soils. Plant Genet. Resour. 15, 221–229. 10.1017/S1479262115000544

[B42] Zhao J. Bayer P. E. Ruperao P. Saxena R. K. Khan A. W. Golicz A. A. (2020). Trait associations in the pangenome of pigeon pea (*Cajanus cajan*). Plant Biotechnol. J. 18, 1946–1954. 10.1111/pbi.13354 32020732 PMC7415775

